# The collagen receptor uPARAP/Endo180 as a novel target for antibody-drug conjugate mediated treatment of mesenchymal and leukemic cancers

**DOI:** 10.18632/oncotarget.17883

**Published:** 2017-05-16

**Authors:** Christoffer Fagernæs Nielsen, Sander Maarten van Putten, Ida Katrine Lund, Maria Carlsén Melander, Kirstine Sandal Nørregaard, Henrik Jessen Jürgensen, Kristian Reckzeh, Kristine Rothaus Christensen, Signe Ziir Ingvarsen, Henrik Gårdsvoll, Kamilla Ellermann Jensen, Petra Hamerlik, Lars Henning Engelholm, Niels Behrendt

**Affiliations:** ^1^ The Finsen Laboratory, Rigshospitalet / Biotech Research and Innovation Center (BRIC), Faculty of Health and Medical Sciences, University of Copenhagen, DK-2200 Copenhagen, Denmark; ^2^ Experimental Animal Models Section, Department of Veterinary Disease Biology, University of Copenhagen, DK-1871 Frederiksberg C, Denmark; ^3^ Proteases and Tissue Remodeling Section, Oral and Pharyngeal Cancer Branch, NIDCR, National Institutes of Health, Bethesda, Maryland 20892, USA; ^4^ Danish Cancer Society Research Center, DK-2100 Copenhagen Ø, Denmark

**Keywords:** uPARAP, antibody-drug conjugate, leukemia, sarcoma, glioblastoma

## Abstract

A key task in developing the field of personalized cancer therapy is the identification of novel molecular targets that enable treatment of cancers not susceptible to other means of specific therapy. The collagen receptor uPARAP/Endo180 is overexpressed by malignant cells in several non-epithelial cancers, notably including sarcomas, glioblastomas and subsets of acute myeloid leukemia. In contrast, in healthy adult individuals, expression is restricted to minor subsets of mesenchymal cells. Functionally, uPARAP/Endo180 is a rapidly recycling endocytic receptor that delivers its cargo directly into the endosomal-lysosomal system, thus opening a potential route of entry into receptor-positive cells. This combination of specific expression and endocytic function appears well suited for targeting of uPARAP/Endo180-positive cancers by antibody-drug conjugate (ADC) mediated drug delivery. Therefore, we utilized a specific monoclonal antibody against uPARAP/Endo180, raised through immunization of a uPARAP/Endo180 knock-out mouse, which reacts with both the human and the murine receptor, to construct a uPARAP-directed ADC. This antibody was coupled to the highly toxic dolastatin derivative, monomethyl auristatin E, via a cathepsin-labile valine-citrulline linker. With this ADC, we show strong and receptor-dependent cytotoxicity *in vitro* in uPARAP/Endo180-positive cancer cell lines of sarcoma, glioblastoma and leukemic origin. Furthermore, we demonstrate the potency of the ADC *in vivo* in a xenograft mouse model with human uPARAP/Endo180-positive leukemic cells, obtaining a complete cure of all tested mice following intravenous ADC treatment with no sign of adverse effects. Our study identifies uPARAP/Endo180 as a promising target for novel therapy against several highly malignant cancer types.

## INTRODUCTION

In many cancer types, notably including several non-epithelial cancers, there is a strong need for novel tumor cell-specific therapy. In these cases, the identification and validation of novel cell surface markers which may act as tumor-specific targets is a crucial task. The urokinase plasminogen activator receptor–associated protein (uPARAP, the product of the MRC2 gene, and also known as Endo180 or CD280) is a specialized component in tissue collagen turnover. This protein, in the following designated uPARAP, acts as an endocytic receptor internalizing extracellular matrix collagen for lysosomal degradation [1–6]. uPARAP has a limited distribution in healthy tissues where it is expressed on a restricted set of cells of mesenchymal origin, including subsets of activated fibroblasts, osteoblasts, and osteocytes involved in bone development [2, 7–10]. In contrast, high expression levels of uPARAP are found on malignant cells of various non-epithelial cancers, including osteosarcomas and soft tissue sarcomas [11, 12], glioblastoma multiforme (GBM) [13, 14] and subtypes of acute myeloid leukemia (AML) (http://servers.binf.ku.dk/bloodspot/, see ref. [15]). In the context of molecular cancer targeting, this is an interesting expression pattern. There is an urgent need for improved means of treatment for all of these cancer types, including a demand for novel strategies for specific tumor cell-directed therapy [16–18].

One successful strategy for targeting of cancer cells through tumor-specific cell surface markers is the use of antibody-drug conjugates (ADCs). This strategy combines the ability of an antibody to specifically recognize the target cell with the effect of an attached drug or cytotoxin, thus allowing targeted drug delivery with minimal side-effects [19–22]. In addition to the strong expression by non-epithelial cancers, uPARAP has functional properties that might particularly favor an ADC-mediated strategy targeting this receptor. Thus, the receptor takes part in efficient ligand internalization in a rapid process, where the receptor recycles to the cell surface with a frequency of several cycles per hour, whereas the initially bound cargo is routed to lysosomal degradation [10, 23].

When targeting such cellular receptors, ADC constructs rely on mechanisms for intralysosomal release of the conjugated cytotoxin from the antibody component, before translocation into the cytoplasm can be efficiently achieved. Furthermore, the limited number of molecular delivery events into each cell dictates the use of extremely potent cytotoxins in these ADC constructs [19]. One commonly employed mechanism of action is based on a highly potent cytotoxin, monomethyl auristatin E (MMAE), a synthetic derivative of the tubulin inhibitor dolastatin 10a [19, 24, 25], which is coupled to an IgG component through a valine-citrulline dipeptide-containing linker entity [26–28]. With this construct, cathepsin-mediated cleavage of the peptide linker in the lysosomal compartment liberates the toxin for entry into the cytoplasm.

In this work, we have constructed and characterized an ADC directed against uPARAP, based on a specific monoclonal antibody in combination with the aforementioned linker-toxin structure. We show that this ADC has strong and target receptor dependent cytotoxicity *in vitro* in a panel of cancer cell lines. Furthermore, we demonstrate a high anti-tumor efficiency *in vivo* in a uPARAP-positive tumor model, obtaining a complete cure in all mice treated with this ADC.  

## RESULTS

### Demonstration of uPARAP-expression in cell lines from various cancers and target-specific endocytosis of antibody 2h9 against uPARAP

**Figure 1:**
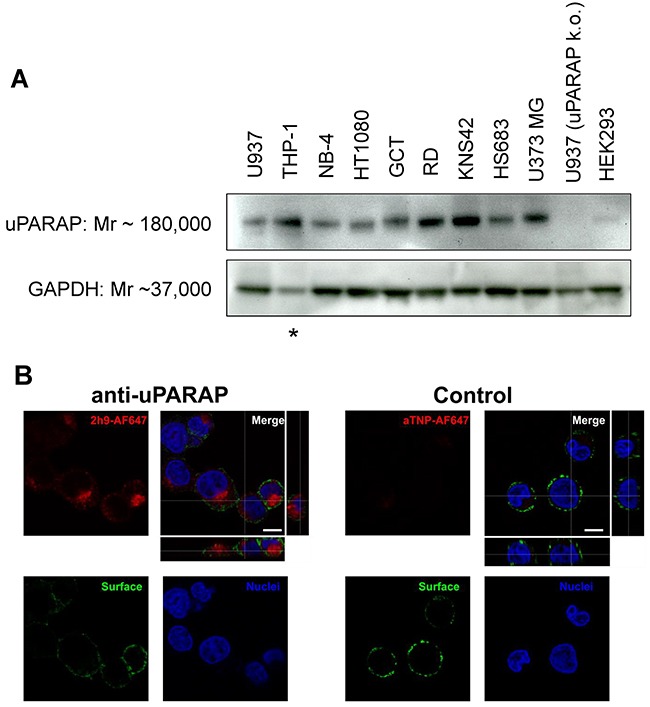
Expression of uPARAP by different cancer cell lines and specific cellular uptake of fluorescence-labeled mAb 2h9 **(A)**, whole cell lysates of the cancer cell lines U937, THP-1, NB-4, HT1080, GCT, RD, KNS42, HS683 and U373 MG were examined for expression of uPARAP by Western blot, together with lysates of uPARAP-deficient U937 cells obtained by CRISPR/Cas9 technology (“U937 uPARAP k.o.”; see Methods) and non-malignant HEK293 cells. uPARAP was detected using anti-uPARAP mAb 2h9 as the primary antibody. Bands of apparent Mr 180,000 confirm expression of uPARAP in all of the cancer cell lines except for U937 uPARAP k.o. Negligible expression of uPARAP is observed in HEK293 cells. An antibody against GAPDH (Mr 37,000) was used as a loading control. Asterisk: The THP-1 cell line displayed a reduced GAPDH signal, although identical loading of total protein was used; see Methods. **(B)**, demonstration of cellular uptake of AlexaFluor 647-conjugated mAb 2h9. U937 cells were incubated with fluorescently labeled uPARAP-directed mAb 2h9 (left) or control mAb aTNP (right), after which the intracellular fluorescence was examined by confocal microscopy. Blue: nuclear (Hoechst) stain; green: plasma membrane (anti-hCD45-AlexaFluor 488) stain; red: AlexaFluor 647-labeled mAbs. z-stack images verify intracellular, vesicular localization of AlexaFluor 647-labeled antibody only in cells incubated with labeled mAb 2h9. Scale bar: 5 μm.

Since uPARAP has been reported to be expressed by tumor cells in AML, sarcomas and GBM [11–15], we first verified that a panel of cultured cell lines derived from these diseases express the receptor. The U937 myeloid leukemia, the NB-4 acute promyelocytic leukemia, the THP-1 monocytic leukemia, the HT1080 fibrosarcoma, the GCT (giant cell tumor) fibrous histiocytoma, the RD rhabdomyosarcoma, the KNS42 glioblastoma, the HS683 glioblastoma and U373 MG glioblastoma cell lines were all found to express uPARAP by Western blotting (Figure 1A). For comparison, we also included a U937 uPARAP knockout cell line rendered uPARAP negative by CRISPR/Cas9 technology (see Methods), as well as the non-malignant, embryonic cell line HEK293, which previously has been shown to have negligible expression of uPARAP [29, 30], and under the same conditions of Western blotting showed a signal close to background (Figure 1A).

As a candidate antibody to be used in ADC form, we focused on a well-defined anti-uPARAP mAb, designated 2.h.9:F12 (henceforth 2h9). This high-affinity antibody was previously raised in the laboratory after immunization of a uPARAP knock-out mouse and has been extensively characterized, including the demonstration of specific reaction with both the human and the murine receptor [29, 30].

To show that mAb 2h9 can accumulate specifically in uPARAP-positive cells, we labeled the antibody with an Alexa Fluor 647 (AF647) fluorophore. For fluorescence labeling, we used a coupling method based on mild reduction of IgG disulphides followed by maleimide conjugation, thus mimicking a preferred molecular strategy for preparing an ADC (see Methods). As a negative control, we labeled an isotype-matched murine mAb, anti-trinitrophenol (aTNP), which has no biological recognition partners in human cells [31]. We then incubated U937 cells with these fluorescence-labeled antibodies, subjected the cells to protease treatment to remove surface-bound fluorescence, and studied the cells by confocal microscopy. When incubated with 2h9-AF647, the cells displayed a pronounced intracellular fluorescence with a strong vesicular signal (Figure 1B). This staining pattern was equivalent to previously obtained results using fluorescence-labeled collagen ligands after specific uptake in uPARAP-positive cells, where these ligands have been shown to be routed to lysosomes [1–5]. No uptake was observed with the negative control, aTNP-AF647. These data suggested that mAb 2h9 could be modified by a reduction/maleimide-dependent conjugation procedure and thereby coupled to a low-molecular weight hydrophobic moiety, while still being subject to specific endocytosis by the target receptor.

### Preparation and evaluation of a uPARAP-directed ADC

**Figure 2:**
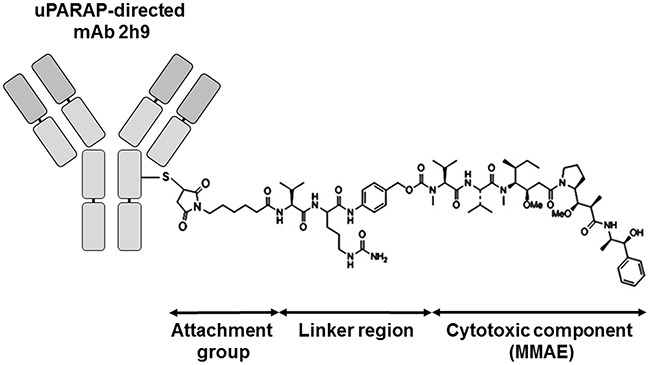
Schematic view of the uPARAP-directed ADC showing the structural constituents of a mAb-vc-MMAE ADC Attachment group: A thiol-directed maleimidocaproyl group. Linker region: valine-citrulline-p-aminobenzoyloxycarbonyl, “vc”. Cytotoxic component: monomethyl auristatin E (MMAE), a potent tubulin inhibitor. The DAR is readily adjustable (see Methods), and a maximal DAR of 10 can be obtained with a murine IgG1 using the employed protocol.

To create an ADC based on mAb 2h9, we coupled the antibody to MMAE via a cathepsin-sensitive linker (see Introduction), using partial reduction of IgG disulphides and maleimide chemistry, forming an ADC designated 2h9-vc-MMAE. An equivalent product was created with the control antibody, aTNP. A schematic view of the resulting mAb-vc-MMAE ADC structure is shown in Figure 2. The molecular drug-to-antibody ratio (DAR) was determined using an absorbance-based method (see Methods), and it was verified that for both of these mAbs, the DAR could be controlled by adjusting the amount of maleimide-derivatized linker-toxin reagent present during conjugation, with a maximal obtainable DAR of 10 [32] (see Methods). This DAR corresponds to labeling of all free thiol groups formed from five reduced interchain disulphides in a murine IgG1 molecule

**Figure 3:**
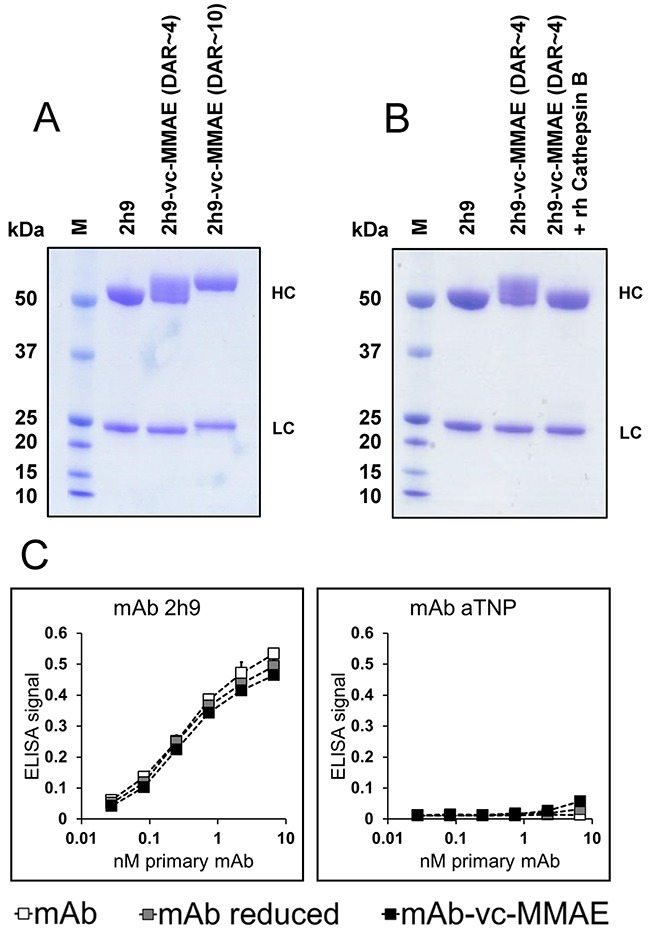
Molecular characterization of ADC products **(A)**, reducing SDS-PAGE of unmodified mAb 2h9, ADC 2h9-vc-MMAE (DAR~4) and ADC 2h9-vc-MMAE (DAR~10). The electrophoretic gel mobilities of the ADC species are reduced with increasing DAR. 2h9-vc-MMAE (DAR~4) is conjugated mainly via the heavy chains (“HC”), whereas 2h9-vc-MMAE (DAR~10) is conjugated via both the heavy and the light chains (“LC”). **(B)**, Reducing SDS-PAGE of mAb 2h9 and ADC 2h9-vc-MMAE (DAR~4) incubated in the absence or presence of recombinant human (rh) cathepsin B. rhCathepsin B reverts the mobility of 2h9-vc-MMAE back to that of mAb 2h9, indicative of linker cleavage and drug release. **(C)** ELISA analysis of unmodified mAbs 2h9 or aTNP (white), reduced mAbs (grey), or mAb-vc-MMAE (DAR~4) ADCs (black), in binding to immobilized uPARAP. 2h9-vc-MMAE displays only negligible loss of affinity towards uPARAP following reduction as well as conjugation.

Batches of 2h9-vc-MMAE with moderate (~4) or high (~10) DAR were analyzed by reducing SDS-PAGE and compared with unmodified mAb 2h9. As shown in Figure 3A, the ADCs have decreased gel mobility relative to the unmodified mAbs and this effect becomes more pronounced at a high DAR. The ADC with a moderate DAR appeared to be mainly conjugated via heavy chain cysteines, evident from the lack of mobility shift of the IgG light chain in the DAR~4 batch. In contrast, the ADC with a high DAR of 10 appeared to be conjugated via both heavy and light chains. To study the sensitivity of the conjugate to proteolytic release of the toxin, we incubated a sample of 2h9-vc-MMAE (DAR~4) with recombinant cathepsin B. This led to a reversion of the electrophoretic mobility back to that of unconjugated mAb, indicative of valine-citrulline dependent linker cleavage by the lysosomal protease (Figure 3B).

To establish that mAb 2h9 retained affinity towards uPARAP after the preparation of the ADC, we studied the binding of the products to uPARAP in an ELISA setup, using dilution series of either unmodified mAbs, mAbs subjected to the reduction step of the conjugation procedure, or mAb-vc-MMAE ADCs. Reassuringly, both the disulphide-reduced mAb 2h9 and the 2h9-vc-MMAE ADC displayed a concentration dependent binding nearly identical with that of the unmodified mAb (Figure 3C). Since the binding curves obtained include the sub-nanomolar concentration range, this result points to an unchanged or negligible loss of affinity towards uPARAP, and confirms that mAb 2h9 tolerates the conjugation procedure with the linker-toxin construct well.

### uPARAP-dependent cytotoxicity of ADC 2h9-vc-MMAE *in vitro*

**Figure 4:**
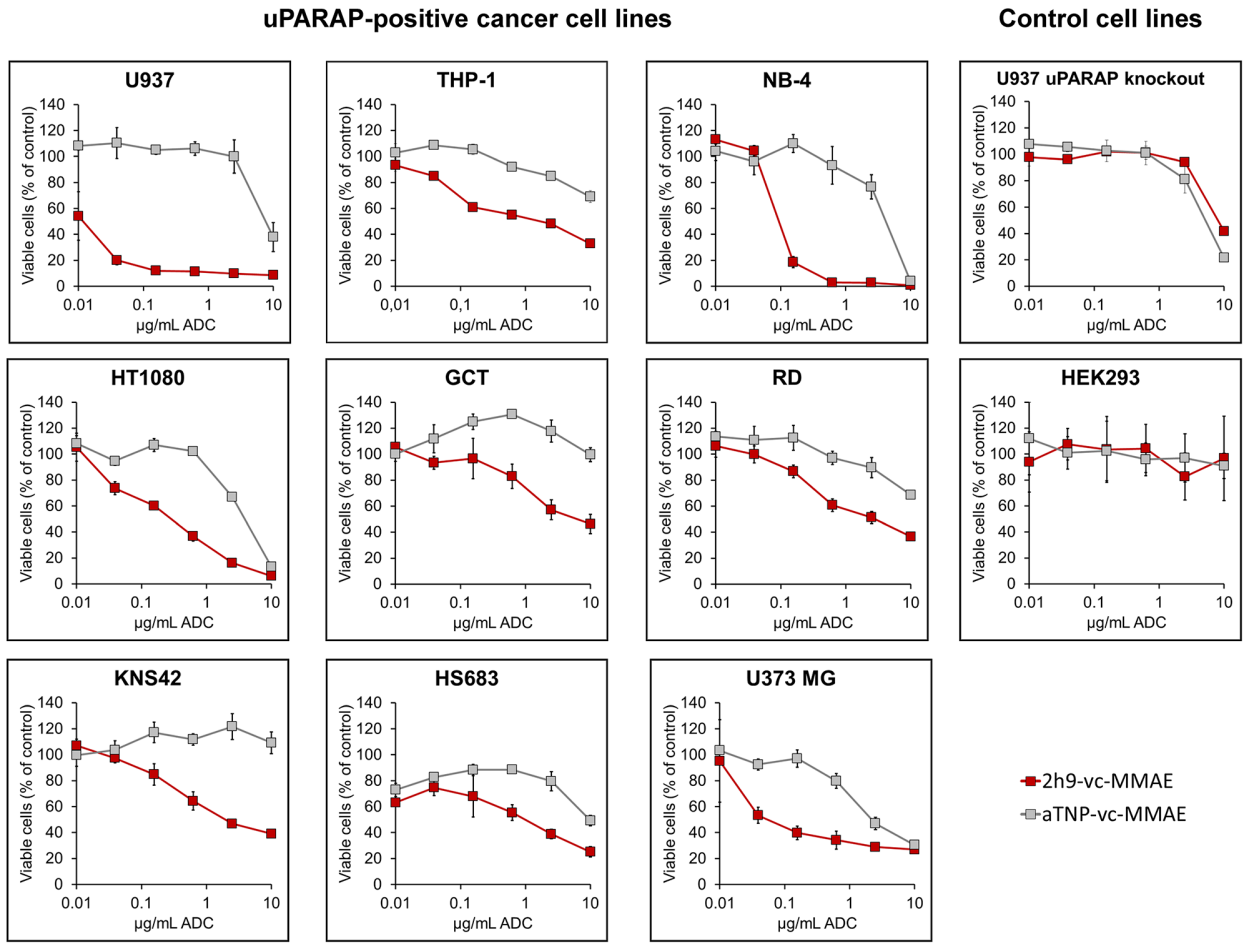
Effect of uPARAP-directed ADC 2h9-vc-MMAE on uPARAP-positive cancer cells *in vitro* MTS-based cell viability assays. Curves display the concentration dependent reduction in cell population viability obtained with uPARAP-directed ADC 2h9-vc-MMAE (red) versus control ADC aTNP-vc-MMAE (grey) in uPARAP-positive U937, THP-1, NB-4, HT1080, GCT, RD, KNS42, HS683 and U373 MG cells, as well as in U937 uPARAP knockout cells and HEK293 cells. Viability is shown as percentage of viable cells, normalized to an untreated control population.

In order to evaluate the cytotoxic potential of uPARAP-directed ADC 2h9-vc-MMAE, cells demonstrated above to be positive or negative for uPARAP expression were incubated with a dilution series of either 2h9-vc-MMAE or control ADC aTNP-vc-MMAE for 72 hours, and thereafter analyzed for cytotoxicity of the ADCs by a cell viability assay (Figure 4). All of the uPARAP-positive cell lines showed specific sensitivity towards 2h9-vc-MMAE, with leukemia cell lines U937 and NB-4 displaying the most pronounced loss of viability. A smaller, nonspecific effect of the negative control conjugate was noted at the highest concentrations (> 2.5 μg/mL mAb component) in some cell lines, but importantly still leaving a large therapeutic window of specificity for 2h9-vc-MMAE. HEK293 cells, as well as the aforementioned U937 uPARAP knock-out clone (see Figure 1A and Methods), were irresponsive towards 2h9-vc-MMAE, thus demonstrating that the cytotoxic effect of this ADC is target receptor specific.

**Figure 5:**
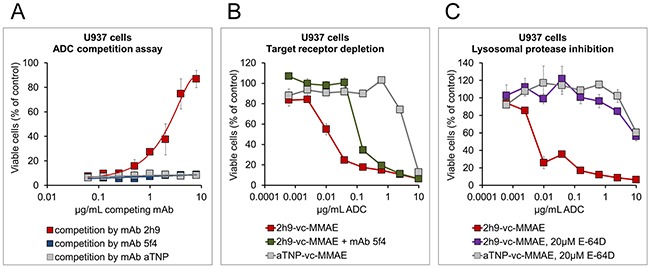
Specificity of the cytotoxic effect of 2h9-vc-MMAE, and importance of cathepsin-mediated linker cleavage MTS-based cell viability assays were performed as in Figure 4, except that additional reagents were added as indicated. **(A)**, receptor competition assay with unmodified mAbs. Viability curves obtained upon incubation of U937 cells with 1 μg/mL of 2h9-vc-MMAE in the simultaneous presence of increasing amounts of potential competitor mAbs, using either mAb 2h9 (red), mAb aTNP (grey) or a different uPARAP-directed mAb (5f4; blue). Only mAb 2h9 rescues the cells from 2h9-vc-MMAE mediated toxicity. **(B)**, effect of target receptor depletion on ADC toxicity. Viability curves of U937 cells obtained with a concentration series of 2h9-vc-MMAE, either without pretreatment of cells (red), following a 3 hour preincubation with uPARAP-depleting mAb 5f4 (green; see text), or obtained with a concentration series of ADC aTNP-vc-MMAE (grey). Preincubation with mAb 5f4 decreases the impact of 2h9-vc-MMAE on cell viability approximately 10-fold. **(C)**, inhibition of lysosomal ADC linker cleavage and drug release. Viability curves of U937 cells obtained in the presence of ADCs 2h9-vc-MMAE (red), aTNP-vc-MMAE (grey), or 2h9-vc-MMAE following preincubation of cells with 20 μM of an inhibitor of lysosomal proteases (E-64D, purple). Incubation in the presence of E-64D abrogates the toxic effect of 2h9-vc-MMAE.

To further substantiate the specificity of the cytotoxic effect, a competition assay was performed. U937 cells were incubated with 2h9-vc-MMAE at 1 μg/mL mAb component in the presence of increasing amounts of potential competitors. The latter reagents included unmodified mAb 2h9, a mAb designated 5f4 which recognizes a different epitope on uPARAP [30], and the negative control mAb, aTNP. The uncoupled mAb 2h9 had pronounced efficacy in rescuing cells from 2h9-vc-MMAE mediated cytotoxicity, whereas no effect was found with the other antibodies (Figure 5A). The mAb 5f4 has been studied extensively previously, and has been shown to deplete uPARAP from the cell surface upon long-term incubation, due to failure of receptor recycling [33]. Therefore, we also studied the effect of this antibody in a preincubation experiment, where mAb 5f4 was added before the addition of 2h9-vc-MMAE. Indeed, preincubating U937 cells with mAb 5f4 for 3 hours resulted in a ~10 fold decrease in the toxicity of subsequently added 2h9-vc-MMAE (Figure 5B).

Next, to study whether the cytotoxicity of 2h9-vc-MMAE was dependent on lysosomal linker cleavage, we tested the effect of E-64D, an inhibitor capable of entering the lysosomal compartment and blocking the activity of several lysosomal cysteine proteases including cathepsin B [34]. U937 cells were preincubated with E-64D, after which the cellular sensitivity was determined by adding a dilution series of the ADCs. As shown in Figure 5C, this pretreatment abrogated the cytotoxic effect of 2h9-vc-MMAE.

Altogether, these experiments showed that the cytotoxic effect of 2h9-vc-MMAE is dependent on the interaction between the targeting antibody and the target receptor, as well as lysosomal cleavage of the valine-citrulline-containing ADC linker.

MMAE is an extremely potent inhibitor of tubulin polymerization and is highly cytotoxic, being directed particularly against actively dividing cells. However, the compound may not always mediate cell killing directly, as affected cells may instead be arrested in the G2/M cell cycle phase due to failure of mitotic spindle assembly [19]. Therefore, in order to investigate the mechanism underlying the observed reduction in overall viability of the cells, we determined the cell cycle distribution of the sensitive cell lines shown above, following incubation with 2h9-vc-MMAE at a concentration resulting in a 50% reduction in viability, as demonstrated in Figure 4. In these experiments, shown in Table 1, all leukemia cell lines (U937, NB-4 and THP-1) displayed a clear propensity towards apoptosis over growth arrest upon exposure to 2h9-vc-MMAE, evident from the increase in percentage of cells mainly in the sub-G1 cell cycle phase. Similar data was found for the sarcoma cell line HT1080, whereas the GCT cell line showed an increase mainly in the fraction of cells in the G2/M phase. The RD sarcoma cell line, as well as GBM cell lines KNS42, HS683 and U373 MG, showed a mixed response, with increased fractions of cells both in the sub-G1 phase and the G2/M phase, indicative of effects resulting in apoptosis as well as growth arrest. Thus, these data demonstrate composite mechanisms of inhibition of cancer cell growth in the cell lines tested. This opens the possibility that both a direct cytotoxicity and a non-lethal inhibition of cell division by the cytotoxin MMAE may contribute to counteracting tumor growth in certain cancer forms.

**Table 1:** Cell cycle phase distribution of uPARAP-positive cell lines after treatment with 2h9-vc-MMAE

Cell line		Sub-G1	G1	S	G2/M
**U937**	Untreated	7,2 ± 0,3	62 ± 0,4	24,3 ± 0,3	6,3 ± 0,3
	2h9-vc-MMAE	**30,0 ± 5,2** *	31,2 ± 1,6	27,6 ± 3,7	**10,8 ± 0,9** *
**NB4**	Untreated	6,2 ± 0,5	49,6 ± 1,1	31,9 ± 0,9	12,2 ± 0,6
	2h9-vc-MMAE	**47,4 ± 1,1** *	15,4 ± 0,0	27,2 ± 1,6	9,4 ± 0,4
**THP-1**	Untreated	3,0 ± 0,2	63,7 ± 0,4	19,3 ± 0,3	14,3 ± 0,9
	2h9-vc-MMAE	**22,0 ± 13,2** *	36,9 ± 17,8	26,3 ± 4,6	14,2 ± 0,7
**HT1080**	Untreated	1,6 ± 0,7	68,7 ± 0,7	9,9 ± 0,6	19,8 ± 1,2
	2h9-vc-MMAE	**22,3 ± 2,0** *	41,2 ± 0,9	17,9 ± 0,6	18,5 ± 1,0
**GCT**	Untreated	8,1 ± 0,3	70,5 ± 1,0	10,6 ± 1,1	10,6 ± 0,9
	2h9-vc-MMAE	9,9 ± 1,0	57,6 ± 0,7	16,0 ± 1,1	**16,4 ± 1,1** *
**RD**	Untreated	2,9 ± 0,4	57,8 ± 1,7	21,4 ± 2,0	17,9 ± 0,7
	2h9-vc-MMAE	**9,2 ± 0,9** *	42,4 ± 2,1	22,7 ± 1,8	**25,6 ± 0,7** *
**KNS42**	Untreated	4,8 ± 0,6	68,9 ± 0,4	14,1 ± 1,2	11,0 ± 1,8
	2h9-vc-MMAE	**35,9 ± 3,0** *	23,3 ± 0,3	15,2 ± 1,4	**23,2 ± 1,8** *
**HS683**	Untreated	6,5 ± 1,7	59,0 ± 0,6	12,5 ± 2,6	22,0 ± 1,9
	2h9-vc-MMAE	**17,8 ± 1,9** *	31,7 ± 0,1	19,7 ± 1,2	**30,8 ± 0,9** *
**U373**	Untreated	4,2 ± 1,0	62,6 ± 1,6	16,3 ± 1,3	15,7 ± 1,7
	2h9-vc-MMAE	**29,5 ± 1,5** *	23,4 ± 1,3	19,6 ± 1,0	**25,6 ± 0,8** *

The cell cycle phase distribution of cancer cell lines, comparing untreated cells to cells following incubation with 2h9-vc-MMAE over three days. The ADC was added at a final concentration resulting in 50% overall cell viability, as previously determined by viability assay (see Figure 4). Bold numbers marked with an asterisk indicate a significant shift in the percentage of cells in a given cell cycle phase (t-test: p<0.01).

### Eradication of uPARAP-positive tumors in mice

The sensitivity of the U937 cell line towards ADC 2h9-vc-MMAE *in vitro* appeared highly promising for *in vivo* investigation, since AML models in mice based on this cell line are well established [35–38], and since uPARAP is strongly upregulated in certain AML subsets in humans [15]. In contrast, normal leukocytes in the circulation are generally uPARAP-negative [6]. Therefore, in order to investigate a potential anti-tumor effect of 2h9-vc-MMAE *in vivo*, we next set up a xenograft model with U937 cells, using subcutaneous inoculation of cells in CB17 SCID mice [35, 36].

**Figure 6:**
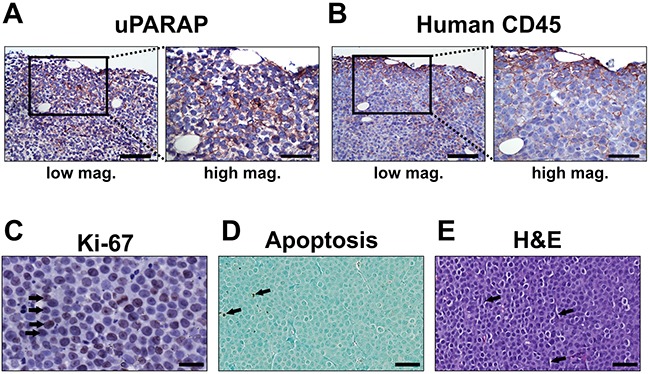
Histology of untreated U937 tumors Histological examination of untreated U937 tumor specimens after inoculation and growth in CB17 SCID mice. **(A and B)**, immunohistochemical localization of uPARAP (A) and human CD45 (B) reveals positive staining of tumor cells (reddish brown), shown at low and high magnification(scale bars 100 μm and 50 μm, respectively). **(C)**, Ki-67 staining of tumor tissue, revealing a high proportion of actively proliferating tumor cells (black arrows). Scale bar: 25 μm. **(D)**, Staining of tumor tissue for apoptosis, demonstrating a very low abundance of apoptotic cells (black arrows). Scale bar: 50 μm. **(E)**, H&E staining of tumor tissue revealed a uniform and dense distribution of tumor cells throughout the entire tumor with relatively few cells with stromal morphology (black arrows). Scale bar: 50 μm.

In this system, the growth of the resulting tumors can be easily evaluated by palpation. In the absence of treatment, subcutaneous injection with U937 cells led to the establishment of local tumors within approx. two weeks, with a doubling time of tumor volume of 3 to 4 days (see Methods; data not shown). Immunohistochemical staining of these tumors revealed a positive staining for uPARAP in tumor cells. uPARAP staining was predominantly observed in the outermost part of the tumor tissue (Figure 6A), which was also the case after positive control staining for human CD45 (Figure 6B), confirming the human origin of the stained cells. Thus, tumor cells retained uPARAP expression when growing *in vivo*. Ki-67 staining of the tumor tissue revealed a high proportion of actively proliferating cells (Figure 6C), as expected considering the rapid tumor growth observed by palpation. Staining for apoptosis showed very little positive signal in the tumor tissue, demonstrating a very low abundance of apoptotic cells (Figure 6D). Evaluation of H&E stained tumor tissue indicated that tumors are densely packed with cancer cells (small round cells), with a relatively small contribution of stromal cells with a typical elongated fibroblast morphology (black arrows). In addition, staining confirmed intact nuclei of the far majority of cells present, with no obvious signs of necrosis (Figure 6E)

To initially test animal tolerance towards the ADCs in the absence of tumors, a subcutaneous injection of a single high dose (15 mg/kg) of either 2h9-vc-MMAE (N=2) or aTNP-vc-MMAE (N=2) at a moderate DAR of ~4 was performed. Animals were then closely monitored for signs of distress or aberrant behavior and weighed daily for a 7-day period. No weight loss or signs of abnormal behavior was observed, thus demonstrating gross tolerance towards these ADCs.

**Figure 7:**
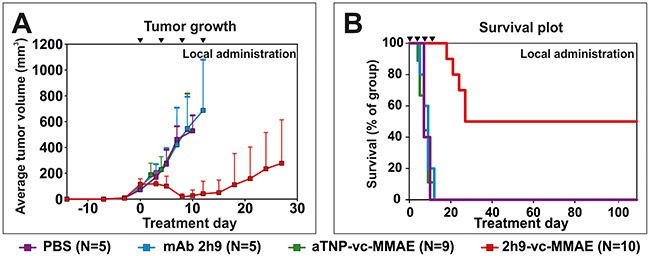
**Treatment of tumors** *in vivo*: **Subcutaneous U937 tumors subjected to local administration of 2h9-vc-MMAE.** Mice were inoculated with U937 cells by subcutaneous injection. Upon the appearance of palpable tumors, local treatment with the indicated reagents was initiated by subcutaneous injection at the tumor site (set as time point day 0). Treatments were given as doses of 3 mg/kg mAb component at day 0, 4, 8 and 12 (black triangles). Treatment groups received 2h9-vc-MMAE (N=10, red), aTNP-vc-MMAE (N=9, green), unconjugated mAb 2h9 (N=5, blue) or PBS (N=5, purple). **(A)**, comparison of the average tumor volume across treatment groups, shown until treatment day 28. **(B)**, Kaplan-Meier plot showing survival of the treatment groups shown in panel A, during an observation period of three months after finishing treatment.

We then tested the effect of local ADC treatment in tumor-bearing mice. Randomized groups of mice were treated with either uPARAP-directed ADC 2h9-vc-MMAE, control ADC aTNP-vc-MMAE, unmodified mAb 2h9, or PBS vehicle control, respectively. Treatment was initiated after the establishment of palpable tumors of 50-100 mm^3^, with each mouse receiving 4 subcutaneous doses of 3 mg/kg mAb component in the tumor area, with four-day intervals. Tumors in mice receiving aTNP-vc-MMAE or unmodified mAb 2h9 showed unaffected tumor growth relative to the PBS vehicle control group, and all mice in these groups reached a point of sacrifice due to tumor size after 9-12 days of treatment. In contrast, the group of mice treated with 2h9-vc-MMAE showed a strong reduction in tumor growth (Figure 7A). Indeed, after completion of treatment, 9 out of 10 mice in this group had reached a point where no palpable tumors could be detected. These mice were then followed for an observation period of 3 months without further treatment. As shown in the Kaplan-Meyer plot (Figure 7B), a full cure was obtained in 5 of these animals, with no signs of relapse throughout this observation period. Notably, these surviving mice did not show any signs of detrimental side effects, referring to the criteria above and general observation of animal behavior. In contrast, 4 of the above 9 mice did show a rapid relapse of tumor growth after termination of treatment. Western blot analysis of tumor tissue revealed that tumors harvested from mice with relapse, as well as tumors from all other treatment groups, were still positive for uPARAP at the point of sacrifice (Supplementary Figure 1). This result pointed to incomplete targeting and elimination of uPARAP-positive tumor cells as being the main reason for the different outcomes for mice in the 2h9-vc-MMAE-treated group, rather than loss of the target receptor during treatment.

**Figure 8:**
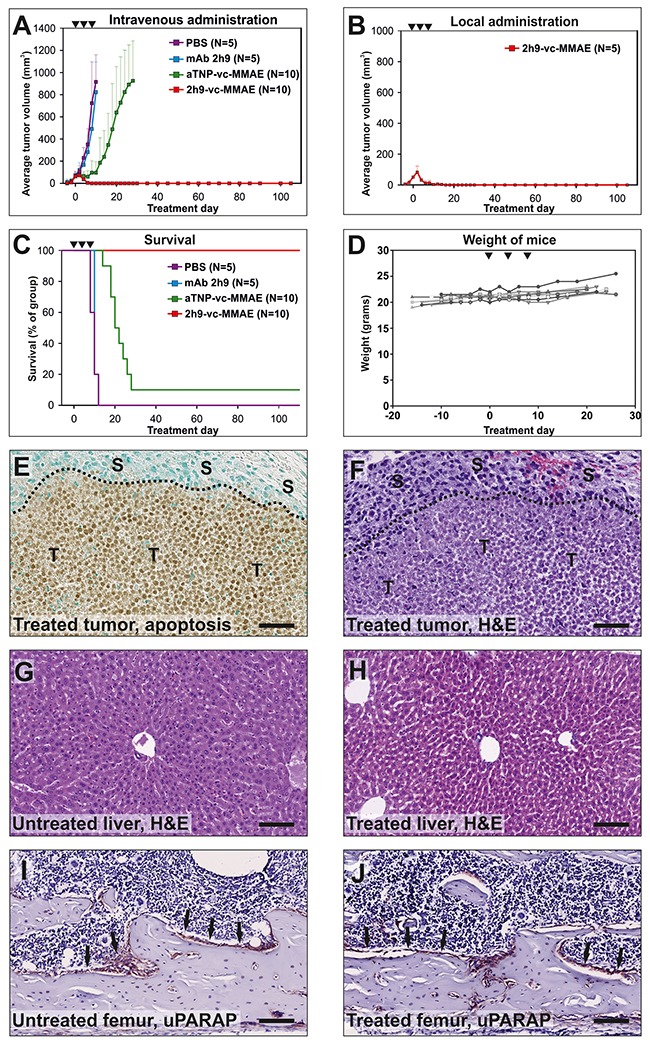
Treatment of tumors *in vivo*: Eradication of subcutaneous U937 tumors by intravenous administration of 2h9-vc-MMAE The experiment was performed as described for Figure 7, except that mice received three doses of 5 mg/kg mAb component by intravenous **(A, C)** or subcutaneous **(B)** injections at day 0, 4, and 8 (black triangles). (A) comparison of the average tumor volume for systemically treated mice across treatment groups. Curves display groups of mice receiving intravenous injections of 2h9-vc-MMAE (N=10, red), aTNP-vc-MMAE (N=10, green), unconjugated mAb 2h9 (N=5, blue) or PBS (N=5, purple). Mice were followed for an observation period of three months after finishing treatment. The curve for the aTNP-vc-MMAE treatment group is discontinued from day 28, when only one surviving mouse remained; this one mouse had no detectable tumor. (B) average tumor volume for a group of mice that received three subcutaneous injections of 2h9-vc-MMAE at 5 mg/kg mAb component at the tumor site (N=5, red). (C) Kaplan-Meier plot showing survival percentages of the treatment groups shown in panel A. **(D)**, individual weight curves of the 10 mice receiving intravenous injections of 2h9-vc-MMAE in panel A, demonstrating stable weight of all treated mice during the treatment period. **(E-J)**, histological examination of tumor and mouse tissue during the process of tumor regression upon 2h9-vc-MMAE treatment. Mice were inoculated with U937 cells as above. After establishment of tumors, mice received a single dose of 2h9-vc-MMAE (5 mg/kg mAb component) by intravenous injection. After four days, tumor shrinkage was confirmed by palpation, mice were sacrificed and histological examination was performed. E, staining of tumor tissue for apoptosis revealing extensive apoptosis of tumor cells upon treatment with 2h9-vc-MMAE, whereas stromal cells are unaffected. “T”: tumor cells, “S”: stromal cells, dotted line: tumor front, scale bar: 50 μm. F, H&E staining of tumor tissue, revealing extensive tumor cell death (little or no nuclear staining), with surrounding viable stromal cells. Symbols and scale bar as in E. G and H, liver sections from untreated (G) or 2h9-vc-MMAE-treated mice (H), revealing no difference after H&E staining. Scale bars: 100 μm. I and J, bone sections (femur) from untreated (I) or 2h9-vc-MMAE-treated mice (J) were immunostained for uPARAP. Sections appeared identical, with prominent uPARAP staining in bone lining cells (reddish-brown, black arrows) of both untreated and treated mice, suggesting minimal target-specific killing of endogenous uARAP-positive cells. Scale bars: 100 μm.

Finally, we tested the effect of systemic treatment with 2h9-vc-MMAE. Mice were inoculated with subcutaneous U937 tumors as above, and after establishment of palpable tumors of 50-100 mm^3^, ADCs were administered intravenously, with a total of 3 injections of 5 mg/kg mAb component in a tail vein with four-day intervals. An additional treatment group was included for comparison, receiving local subcutaneous treatment with 2h9-vc-MMAE as above, but with the same dose and treatment schedule as the groups receiving intravenous treatment. Remarkably, intravenous treatment with 2h9-vc-MMAE resulted in a complete cure of all mice, with no recurrent tumor growth over a 3 month observation period (Figure 8A). The same outcome was obtained after local subcutaneous treatment with this slightly elevated dose of the ADC (Figure 8B).

Survival of the intravenously treated mice is shown in a Kaplan Meier plot (Figure 8C). Although tumors were not completely inert to intravenous treatment with control ADC aTNP-vc-MMAE (Figure 8A and 8C), the effect noted in this group was much less pronounced than that obtained with 2h9-vc-MMAE, and most likely was the result of an enhanced permeability and retention (EPR) effect (see Discussion).

The group of mice treated systemically with 2h9-vc-MMAE was followed throughout the treatment and a 3 month observation period. The mice showed no weight loss during the treatment period (Figure 8D), and observations revealed no negative side effects in terms of aberrant animal behavior.

A separate group of U937 tumor-bearing mice was then treated with a single intravenous injection of 2h9-vc-MMAE at 5 mg/kg, and upon tumor shrinkage confirmed by palpation, these tumors were resected and subjected to histological analysis. Staining of the tumor tissue for apoptotic cells revealed an extensive positive staining across the entire tumor, indicative of efficient targeting of tumor cells by 2h9-vc-MMAE, with the surrounding stromal tissue being largely negative for apoptosis (Figure 8E). This pattern of extensive cell death in the tumor tissue, with cells of the tumor stroma remaining unaffected, was confirmed by H&E staining (Figure 8F).

To further study potential side effects, we examined the histology of mouse livers during ADC treatment for any signs of acute, non-specific hepatotoxicity. H&E staining of liver sections of treated mice, obtained at the same time point as above where tumor regression was evident, revealed no histological abnormalities [39, 40], and no differences between untreated and treated livers could be noted (compare Figure 8G and 8H). Furthermore, to look into the fate of healthy cells that do express uPARAP, we focused on bone lining cells that are well documented to express the receptor [41]. At the time of tumor regression, bones of treated mice were indistinguishable from those of untreated mice, and bone lining cells were in both cases positive for uPARAP expression (compare Figure 8I and 8J).

Taken together, these results demonstrate highly efficient tumor penetration and targeting of tumor cells by ADC 2h9-vc-MMAE. Additionally, uPARAP-positive host cells appeared to be unaffected, adding to the tumor specificity of this ADC.

## DISCUSSION

In this work, we have identified the collagen receptor uPARAP as a novel and highly promising target for specific drug delivery to different types of non-epithelial cancers. Cancer cells positive for this receptor are dominant in sarcomas [11, 12], GBM [13, 14] and subtypes of AML [15], and uPARAP positive cell lines derived from these cancers were demonstrated to be sensitive *in vitro* to our uPARAP-directed ADC in a target receptor specific manner (Figure 4, Figure 5 and Table 1). Treatment experiments *in vivo* with transplanted U937 cells in mice led to complete eradication of established tumors (Figure 8).

The use of ADCs for targeted drug delivery is a rapidly growing approach, with the number of new clinical trials on ADC drugs having increased steadily for the last decade. As of late 2016, almost 100 different ADC species had undergone clinical testing in various forms of cancer, with about 60 ADCs in active testing, and more than 100 preclinical ADCs underway [42]. The process of developing these potential drugs has led to substantial understanding of factors critical for a successful ADC strategy. Notably, this includes the utilization of extremely cytotoxic agents which, in their free form, would be much too toxic to be safely used for cancer therapy, as well as the introduction of molecular properties that make the ADCs non-toxic before specific release of the conjugated toxin inside the target cell [19, 22].

The linker-toxin entity used in our work, vc-MMAE, was originally developed in pioneering studies to create the ADC Brentuximab Vedotin (Adcetris®), directed against CD30-positive cancers [26, 27, 43, 44]], and since then, vc-MMAE has been widely used in ADC development [21, 42]. Although the functional properties of a combination of a certain IgG molecule and any linker-toxin construct is unpredictable, we showed that the mAb 2h9 directed against uPARAP could be efficiently coupled to vc-MMAE using a reduction/maleimide conjugation method, with negligible loss of affinity towards the target receptor. The resulting uPARAP-directed ADC was proven to be efficacious against a panel of uPARAP-positive cancer cell lines with negative control cells being unaffected, demonstrating uPARAP-dependent cytotoxicity.

The current combination of molecular target and use of an ADC strategy has several advantageous properties. In addition to the unique cellular expression pattern, the targeted molecule uPARAP is an efficiently recycling endocytic component, belonging to the clathrin-associated group of constitutive internalization receptors with direct lysosomal delivery of cargo [1–6]. These endocytic properties are rarely found among existing ADC targets in clinical testing [21, 42] and may likely contribute to a high efficiency of ADC internalization, which in turn may favor efficacy of treatment with this type of ADC. In contrast, ADC targeting of cell surface proteins which are not bona fide internalization receptors has often required the development of specialized antibodies, which actively induce the internalization of these targets [45], or even more complex bispecific antibody formats, in order to obtain efficient ADC internalization [46].

While our ADC was highly specific in killing and/or inducing growth arrest in uPARAP-positive tumor cells *in vitro*, there appeared to be no simple correlation between the degree of sensitivity and the apparent levels of uPARAP expression in the target cells (compare Figure 1A, Figure 4 and Table 1). A similar situation has often been reported for other ADCs in pre-clinical studies, where ADC cytotoxicity does not necessarily reflect the target receptor expression level in a simple linear manner [26, 47, 48]. Although a high expression level of any target receptor would be considered beneficial in regards to ADC efficacy against a certain type of cancer cell, there is a general consensus that several additional factors influence the overall sensitivity towards a given ADC species [19–22]. Notably, these factors include a variable sensitivity towards the cytotoxin component of the ADC, which in turn may be influenced by the expression of multidrug resistance transporters [49] and, in the case of tubulin inhibitors such as MMAE, the propensity to cell division. Other factors may include the overall internalization and routing rates of ADCs [50, 51], as well as the cellular capacity for linker cleavage. It is not unlikely that all of these factors contribute to the variations in sensitivity observed in our panel of cancer cell lines (Figure 4 and Table 1). In particular, we demonstrated that ADC efficiency in highly sensitive cells such as U937 cells is completely dependent on lysosomal linker cleavage, since uPARAP-dependent cytotoxicity was abrogated after inhibition of lysosomal proteases with E-64D (Figure 5C).

The mAb 2h9 was obtained after immunization of a uPARAP gene-deficient mouse and is reactive with an epitope shared between human and murine uPARAP [29, 30]. This leads to a great advantage when using xenograft tumor models in mice, since treatment with a 2h9-based ADC will not only reveal a successful targeting of human tumor cells, but also any potential target-dependent toxicity in the host organism. We found ADC 2h9-vc-MMAE to be well tolerated at high doses in mice, and no side-effects were observed in the three month observation period following successful treatment. This is not unexpected in the light of the known expression pattern of this receptor. Both in humans and mice, although uPARAP is found in a variety of organs, the expression is restricted to a limited set of activated, mesenchymal cell types in the healthy adult organism, typically in connection with tissue remodeling (reviewed in [6]). Although a detailed toxicology study was not performed in this work, our results suggest that healthy uPARAP-positive host cells are not particularly sensitive to 2h9-vc-MMAE. A prominent example of healthy, uPARAP-expressing cells is bone lining cells which take part in bone collagen metabolism [41]. However, these cells were intact and retained uPARAP expression during ADC treatment (Figure 8I and 8J), even though at the same time point, tumor regression and extensive apoptosis of tumor cells was evident (Figure 8E and 8F). Most likely, the resistance of bone lining cells towards 2h9-vc-MMAE is due to factors such as a low mitotic activity discussed above, which further contribute to narrow the specificity of treatment to uPARAP-expressing tumor cells. Regarding generalized (non-target related) activity of ADCs, this is often revealed in the form of hepatotoxicity, with necrosis and inflammation being evident upon simple inspection of liver sections as found in mouse studies [39, 40]. However, no sign of liver damage was observed upon treatment with 2h9-vc-MMAE (Figure 8G and 8H).

In our xenograft tumor model with U937 cells in CB17 SCID mice, intravenous administration of 2h9-vc-MMAE led to a 100% cure rate of the treated mice. By far the major element in this effect was a uPARAP-dependent cytotoxicity, as evident from a comparison of the four treatment groups (Figure 8A and 8C). Although the detailed mechanism of cell death was not pursued *in vivo*, immunohistochemical studies performed on 2h9-vc-MMAE-treated tumors during the regression phase showed that a single injection of 2h9-vc-MMAE led to widespread and specific death of cancer cells, while surrounding stromal cells remained unaffected by ADC treatment (Figure 8E and 8F).

In addition to uPARAP-specific eradication of tumors, a slight unspecific effect was also noted *in vivo*, with the non-targeted control ADC, aTNP-vc-MMAE, displaying a much lower but still measureable effect on tumor growth (Figure 8A and 8C). Similar observations have been done in other ADC studies, including investigations that employ the vc-MMAE linker-toxin construct with different targeting antibodies [27, 48, 52–55]. The unspecific contribution to such an anti-tumor effect is usually ascribed to an EPR phenomenon, resulting in accumulation of macromolecular drugs in the tumor microenvironment due to leaky vasculature and a lack of lymphatic clearance, as well as extracellular cleavage and/or degradation of ADCs in the intratumoral area [56, 57]. To this end, additional studies have shown that non-internalizing ADCs, based on more labile linker chemistries, can even in some cases contribute to treating solid tumors by passive diffusion of released cytotoxins [58, 59].

In this work we utilized a well described AML xenograft model to show the efficiency of our uPARAP-directed ADC *in vivo*. However, the potential therapeutic utilization may cover several additional types of cancers. The expression patterns described above would point directly to sarcomas and glioblastoma as likely indications, i.e. very severe diseases for which only few options for targeted therapy exist [16–18]. Indeed, we have recently studied the expression and importance of uPARAP particularly in osteosarcoma, where just the functional inhibition of this receptor leads to a pronounced counteraction of tumor-mediated bone degeneration in a mouse model [11]. In connection with treatment of this disease, it is particularly promising that we found healthy, uPARAP-positive bone lining cells to be tolerant to systemic ADC treatment in the current study.

In addition to these cancer forms, uPARAP displays a strong upregulation in the stromal compartment of several cancers of epithelial origin, including breast [60–62], head and neck [30] and prostate cancer, where even a mixed tumor-stroma expression has been reported for the latter [63, 64]. Along with an increasing understanding of the functional importance of the tumor microenvironment, an increasing interest is now devoted to therapeutic targeting of stromal cells in cancer [65–68], as well as bystander effects utilizing stromal liberation of the toxin components of ADCs. In this connection, our uPARAP-directed ADC may also be a highly efficient reagent, due to the cell permeability of the liberated MMAE toxin.

In all potential indications for treatment with a uPARAP-directed ADC, the complete target specificity is a great advantage, not only for reducing side effects but also for the option of selecting individual patients for treatment. Most of the diseases discussed above are highly heterogeneous in their protein expression pattern and therefore, an immunohistochemical examination of primary tumor material should strongly aid the identification of patients with a dominant expression of the target protein.

The linker-toxin combination employed in this study, vc-MMAE, is currently the most widely employed construct in the ADC field [22, 42]. However, novel ADC functionalities are continuously being developed. These include improved and site-directed attachment chemistries, improved linkers, and especially in current years more potent cytotoxins [19–21, 25, 69]. These innovations all contribute to a continuously increasing perfection of ADC synthesis and function, while studies such as the one presented here should potentially contribute to expanding the scope of ADC utilization in preclinical as well as clinical settings via the identification of novel targets.

We hope that the novel findings presented in this work will pave the way for a further development of uPARAP-directed ADCs, and ultimately lead to actual cancer therapy based on the concepts introduced in this study. This may be an important contribution to combatting cancer types with an urgent need for novel means of treatment.

## MATERIALS AND METHODS

### Antibodies and cell lines

uPARAP-directed mAbs 2h9 and 5f4 of isotype IgG1k, as well as the isotype-matched control mAb directed against trinitrophenol, aTNP, were produced at the laboratory using hybridoma cell cultures as described previously [30, 31, 70, 71]].

U937 (ATCC# CRL1593.2), THP-1 (ATCC# TIB-202), HT1080 (ATCC# CCL121), GCT (ATCC# TIB-223), RD (ATCC# CCL-136), HS683 (ATCC# HTB-138), U373 MG (ATCC# HTB-17) and HEK293 (ATCC# CRL-1573) cells were all obtained from ATCC. KNS42 cells were kindly provided by Drs. Lara Perryman and Janine Erler, Biotech Research and Innovation Centre (BRIC), University of Copenhagen. NB-4 cells were kindly provided by Dr. Kim Theilgaard-Mönch, The Finsen Laboratory, Copenhagen University Hospital.

A U937 cell line, deficient for uPARAP expression for use as a negative control, was prepared by CRISPR/Cas9 technology using previously published detailed protocols [72]. In brief, a human uPARAP sgRNA1 (5′-GCCGAAACCGGCTATTCAACCTGG-3′) was designed to target exon 2 of the uPARAP gene, corresponding to the Cysteine-rich domain in the N-terminal part of the protein. After sgRNA1 cloning into the plasmid pSpCas9(BB)-2A-GFP, the resulting plasmid was used for transfection of U937 cells using Lipofectamine 3000. Positively transfected cells were selected using FACS enrichment of GFP expressing cells followed by single cell sorting into 96-well plates [73]. Single clones were screened for the absence of uPARAP expression by flow cytometry in 96-well format using anti-uPARAP mAb 5f4 for staining, and clones with a negative staining signal in comparison to wild-type control cells were further propagated in cell culture. uPARAP deficiency was further confirmed by Western blotting using anti-uPARAP mAb 2h9. Genomic DNA of selected clones was purified and subjected to PCR amplification of the region surrounding the expected Cas9 target site by use of forward (5′-AACGTATGAGTGACGGCTCA-3′) and reverse (5′-CTCCTCGCTGCCGTAGATG-3′) PCR primers. To determine indels and DNA sequences of knockout clones, the PCR-products were first purified by QIAQuick PCR purification before direct Sanger sequencing followed by TIDE and “mixed sequence reader” analysis [74, 75]. To this end, the uPARAP knockout (−/−) monoclone 2A3 used herein, was determined to contain a targeted 1 nt insertion (A) and a 4 nt deletion (TTCA), respectively, in the uPARAP-encoding alleles.

Cell lines were maintained in the following media: unmodified U937, U937 clone 2A3, THP-1 and NB-4 in RPMI; HT1080, RD, U373, HS683 and HEK293 in DMEM; KNS42 in DMEM/F-12, and GCT in McCoy's 5A medium. All media were supplemented with 10% fetal bovine serum and 1% penicillin/streptomycin, and cells were maintained in a 37°C, 5% CO_2_ atmosphere incubator.

### Western blot for detection of uPARAP expression in cell lysates

For preparation of cell lysates, cells were treated on ice for 20 min with lysis buffer (10 mM Tris, 140 mM NaCl, 1% Triton X-100, pH 7.4, including protease inhibitor cocktail set III EDTA-free (Calbiochem) diluted 1:200), using 100μL of lysis buffer per 1×10^6^ cells, then centrifuged at 20,000 x G. Sample protein content was measured by Pierce BCA analysis (ThermoFisher). 25 μg of total protein per sample was then run on a 4-12% gradient gel using the NuPAGE Bis-Tris SDS-PAGE gel and reagent system (ThermoFisher Scientific). Using an iBlot transfer system (Invitrogen), gel contents were transferred to a PVDF membrane using a Novex PVDF gel transfer stack (Invitrogen). The membrane was then blocked in 2% BSA in PBS pH 7.4 for 30 minutes.

Membranes were then incubated in primary antibody (anti-uPARAP mAb 2h9 at 0.5 μg/mL or anti-human GAPDH mAb #VMA00046 from Bio-Rad (loading control) 1:2000), diluted in PBS with 0.1% Tween 20, pH 7.4, followed by HRP-conjugated secondary rabbit anti-mouse antibody 1:6000 (#P0260, Dako Denmark), and treatment for 5 minutes with Amersham ECL Western Blotting Detection Reagent (#RPN2106, GE Healthcare), with intermediate washing steps. The blot was developed by a brief exposure onto Amersham Hyperfilm ECL (#28906837, GE Healthcare).

In these analyses, the THP-1 cell line displayed a reduced GAPDH signal, although identical amounts of total protein were analyzed and loaded as measured by Pierce BCA analysis (Figure 1A). A parallel gel stained with Coomassie Blue confirmed a uniform protein loading for all cell lines analyzed (Supplementary Figure 2).

### Analysis of cellular uptake of fluorescence labeled mAb against uPARAP

Fluorescently labeled antibodies 2h9 and aTNP were prepared by subjecting the antibodies to a mild reduction with 10 mM DTT in PBS, pH 7.4 for 30 minutes at 37°C, followed by buffer exchange to fresh PBS pH 7.4 using a 30 kDa NMWL Amicon Ultra Centrifugal Filters (Merck Millipore). Antibodies at 2 mg/mL were then allowed to react with a 5-fold molar excess of Alexa Fluor 647 C2 Maleimide (AF647, Thermo Fischer Scientific) for 2 hours at room temperature, and then purified by gel filtration on PD-10 desalting columns (GE Healthcare Life Sciences).

For analysis of cellular uptake of antibodies, cells were incubated for 4 hours with AF647-labeled mAbs at 5 μg/mL. A nuclear Hoechst stain (#62249, Thermo Fisher) was added at 2 μg/mL in cell medium for 20 minutes before washing, followed by treatment with a mixture of 0.25% trypsin-EDTA (Gibco, Life Technologies) and 50 μg/mL proteinase K (Roche) in order to remove surface-bound antibodies. For staining of the plasma membrane, an anti-human CD45 mAb (#368502, BioLegend) was fluorescence-labeled using a Zenon Alexa Fluor 488 Mouse IgG1 Labeling Kit (#Z25002, Thermo Fisher Scientific), then added to the cells at 5 μg/mL in PBS for 20 minutes. Cells were then spun down onto a microscope glass slide (Superfrost ultra plus, Thermo Scientific) using a Hettich cyto-system at 500xG for 1 minute in 4 mL one-funnel chambers (#M966-4, Simport, Canada), and mounted in ProLong Gold (Thermo Fisher Scientific) under a cover slide. Samples were then examined using a Leica TCS SP8 confocal laser microscope.

### Preparation and characterization of mAb-vc-MMAE ADCs

For preparation of ADCs, antibodies were subjected to mild reduction by a 15 minute incubation at 37°C in the presence of 10 mM DTT in a 50 mM sodium borate, 50 mM NaCl, pH 8.0 buffer at an IgG concentration of 5 mg/mL, followed by buffer exchange to fresh PBS pH 7.4 with 1 mM EDTA, using 30 kDa Ultra Centrifugal Filters as described above. This was followed by immediate conjugation by addition of a molar excess of maleimidocaproyl-valine-citrulline-p-aminobenzoyloxycarbonyl-monomethyl auristatin E (MC-VC-PAB-MMAE, Levena Biosystems, San Diego, USA), dissolved in water-free DMSO to a final DMSO content of 10% v/v during conjugation for 2 hours at 37°C. Amounts of MC-VC-PAB-MMAE were adjusted according to the desired DAR; see below. The resulting mAb-vc-MMAE ADCs were purified by gel filtration on PD-10 desalting columns (GE Healthcare Life Sciences). The average DAR of the resulting ADCs was measured based on the absorbance ratios of purified conjugate preparations at λ=248nm (A_max_ of MMAE) and λ=280nm, respectively, using previously determined reference values [43, 76]. With the coupling method employed, conjugation of MC-VC-PAB-MMAE at 5-fold molar excess over mAb resulted in moderate DARs of 4-5, whereas conjugation at 10-fold molar excess resulted in a DAR of about 10. For cytotoxicity studies *in vitro*, ADCs with a DAR~10 were employed. Studies *in vivo* were performed using ADCs with a DAR of 4-5.

### Electrophoretic analysis of conjugate species and test of linker cleavage

SDS-PAGE was performed with 5 μg of total protein per lane, using the same gel system as described above for Western blotting. Samples were reduced by boiling for 3 minutes in sample buffer in the presence of 40 mM DTT before electrophoresis. A protein marker was run in separate lanes for Mr determination. Gels were stained using a standard 0.1% Coomassie blue stain. For linker cleavage assays, samples of mAb-vc-MMAE were treated with recombinant human (rh) Cathepsin B (#953-CY, R&D systems) according to the manufacturer's instructions, using 100 ng of activated rhCathepsin B to 20 μg mAb or ADC (mAb component) in a 25 mM 2-(N-morpholino) ethanesulfonic acid (MES) buffer, pH 5.0, and incubation at 37°C overnight.

### ELISA of uPARAP-binding by conjugated mAbs

A 96-well ELISA plate was coated with 25 ng/well of a soluble truncated uPARAP protein comprising the first 3 domains of uPARAP, containing the epitope for mAb 2h9 [29, 30]. Dilution series of untreated mAbs (2h9 or aTNP), partially reduced mAbs (see above), or ADCs 2h9-vc-MMAE or aTNP-vc-MMAE, were then employed as the primary antibody in an ELISA setup, followed by an HRP-conjugated rabbit anti-mouse Ig secondary antibody (Dako Denmark). Finally, an o-phenylenediamine dihydrochloride (OPD) ELISA substrate solution (Dako) was added, and the color reaction was stopped by adding 1M H_2_SO_4_. Plates were read at 492 nm using a plate reader.

### Cytotoxicity of 2h9-vc-MMAE *in vitro* - Cell viability assay and cell cycle analysis

For cell viability assays, cells were seeded at low density, generally 1×10^3^ to 2.5×10^3^ cells per well, in a flat-bottomed 96-well cell culture plate (Costar) in 90 μL of cell culture medium. The next day, mAb-vc-MMAE conjugates were prepared as a serial dilution (1:4) in PBS and added in volumes of 10 μL to each well, with a maximum final ADC concentration of 10 μg/mL mAb component in cell culture medium with 10% v/v PBS. Cells were incubated for 72 hours before evaluating overall viability by adding 12 μL of CellTiter 96 AQueous One Solution Cell Proliferation Assay (MTS) (Promega), and then further incubating for an appropriate time for formation of color (15-60 mins). The plates were then read at 490 nm, with background subtraction at 630 nm using a plate reader, and viability calculated as percentage of an internal plate control of untreated cells. For cell cycle analyses, cells were seeded and treated with 2h9-vc-MMAE as above, at a concentration yielding about 50% viability in the abovementioned MTS viability assays. After 3 days, cell cycle analysis was performed using a Nucleocounter NC-3000 system (ChemoMetec Denmark), according to the manufacturer's standard protocol for analyzing the cell cycle distribution in a population of cells, based on DNA content. The percentage of cells in Sub-G1-, G1-, S-, or G2/M-phases of the cell cycle was established from histogram analysis using the NucleoView NC-3000 software.

### Receptor competition assay, receptor depletion assay and lysosomal protease inhibition

These experiments were done in the form of cell viability assays with U937 cells, performed as above, except that additional reagents were added, either in a pre-treatment step or at the start of ADC treatment of cells. Receptor competition assay was performed with a constant 2h9-vc-MMAE concentration of 1 μg/mL mAb component in all wells, and with the ADC and the unmodified competing mAb being added simultaneously. Competing mAbs were used in dilution series (1:2), starting at 8 μg/mL and were present throughout the assay. For receptor depletion assay, cells were pre-incubated in the presence of mAb 5f4 at 10 μg/mL for 3 hours, before starting a 72 hour cell viability assay in the presence of 2h9-vc-MMAE as described above. For the inhibition of lysosomal proteases, U937 cells were pre-incubated with 20 μM of E-64D protease inhibitor (Sigma-Aldrich) for 2 hours, before starting a 72 hour cell viability assay in the same manner.

### Animal experiments

All animal experiments were performed under legal approval from The Danish Veterinary and Food Administration, under license number 2014−15−0201−00322 (L. H. Engelholm). All reagents and cell lines used for animal experiments were tested negative for the presence of endotoxins, murine viruses, bacteria, mycoplasma and fungi (Taconic Europe, Ry, Denmark). For all studies, animals received standard of care, according to Danish animal welfare legislation.

A xenograft model with subcutaneous inoculation of human U937 cells was established in CB17 SCID mice (Janvier Labs). Mice were injected subcutaneously in the upper right flank with 1×10^6^ U937 cells in 100 μL PBS, resulting in the formation of tumors displaying rapid growth, with an observed doubling in tumor volume of approximately 3-4 days. Tumor growth was followed by palpation using electronic calipers. Volumes were calculated using the formula V=(LxW^2^)/2, with L being the longest dimension of the tumor and W being the corresponding tumor width.

Studies on treatment of subcutaneous tumors were performed with either local (subcutaneous) or systemic (intravenous) administration of reagents. For the study on local administration, upon formation of palpable tumors with a volume of ~50-100 mm^3^, mice were randomized into four treatment groups, receiving either 2h9-vc-MMAE (N=10), aTNP-vc-MMAE (N=9; one mouse in a group of 10 failed to develop palpable tumors), unmodified mAb 2h9 (N=5) or PBS vehicle control (N=5). All treatments were given as a total of four subcutaneous doses of 3 mg/kg mAb component near the tumor, with one dose every four days (q4dx4 schedule). Injections were performed under brief isoflurane anesthesia. During treatment, the tumors were measured every second day, until reaching a point of sacrifice (euthanasia due to tumor burden). Mice which completely lost any tumor burden were then examined twice a week for a period of 3 months after the final injection.

For the study on systemic treatment, mice were inoculated with U937 cells as above, again allowing tumors to reach a volume of ~50-100 mm^3^ before initiation of treatment. The four treatment groups received either 2h9-vc-MMAE (N=10), aTNP-vc-MMAE (N=10), unmodified mAb 2h9 (N=5) or PBS vehicle control (N=5). All treatments were given as a total of three intravenous doses of 5 mg/kg mAb component in the tail vein, every four days (q4dx3 schedule). During treatment, tumors were measured every second day, until reaching a point of sacrifice (euthanasia due to tumor burden). Mice which completely lost any tumor burden were examined twice a week for a period of 3 months after the final injection.

Treatment experiments were performed with the researcher responsible for injecting mice and evaluating tumor size being blinded from the identity of the treatment groups.

### Harvest and processing of tissue for immunohistochemical staining

Tumor tissue was harvested from tumor-bearing mice upon reaching a point of sacrifice: Mice received anesthesia (Hypnorm/Midazolam), and the mice were perfused by intracardial injection of 10 mL PBS. The tumors were then surgically removed and cut in half, with one half being immediately frozen in a mixture of dry ice and ethanol for Western blot analysis of uPARAP expression, and one half being transferred to a 4% PFA solution for histological processing. Bones were dissected and transferred to a 4% PFA solution in a similar manner. After 24 hours of incubation at 4°C, tissue samples for histology were transferred to 70% ethanol. Bones were decalcified in a 10% water solution of EDTA, adjusted to pH 7.4, by microwave heating steadily to 50°C for 20 minutes at 600W, then maintaining 50°C for 2 hours at 300W. To ensure complete decalcification, the femurs were stored in fresh 10% EDTA, pH 7.4, for 3 days at 4°C. Samples for histology were then dehydrated with 70%, 96% and 99% ethanol, followed by xylene, and subsequently embedded in paraffin. The embedded tissue samples were sectioned at 3-3.5 μm on an automatic microtome (#HM355S, Thermo Scientific), and mounted on glass slides.

### Immunohistochemical staining of tissue samples

*uPARAP and hCD45 staining*: Samples were deparaffinized and subjected to a pretreatment with proteinase K at 5 μg/mL (uPARAP) or 10 mM citrate buffer, pH 6.0 (hCD45), as well as blocking of endogenous peroxidase by 1% H_2_O_2_, before incubation with primary detection antibody (anti-uPARAP: rabbit-anti-human pAb [2] at 0.5 μg/mL, anti-hCD45: #368502, Biolegend, at 1 μg/mL) overnight at 4°C. Envision-HRP anti-rabbit (#4003, Dako) or Envision-HRP anti-mouse (#K4001, Dako) was employed as a secondary antibody, respectively, and NovaRED HRP substrate (#SK-4800, Vector Burlingame) was added as a color substrate for 9 minutes before rinsing and adding a hematoxylin counterstain (HARRIS HTX solution, #01800, HistoLab). Samples were finally dehydrated with increasing concentrations (70%, 96% and 99%) of ethanol, followed by xylene, and mounted with Pertex (#20080, Sakura Finetek). *H&E staining*: Samples were deparaffinized, rinsed in water, and subjected to a staining with hematoxylin (HARRIS HTX solution, #01800, HistoLab) for 5 minutes followed by staining with eosin (eosin solution 0.2%, #8702, Sakura Finetek) for 5 minutes. Samples were finally dehydrated with increasing concentrations of ethanol followed by xylene and mounted with Pertex, all being performed as above. *Apoptosis staining*: Staining for apoptotic cells was performed using the In situ Apoptosis Detection Kit (#ab206386, Abcam), according to the enclosed manual. Briefly, samples were first deparaffinized and subjected to a pretreatment with proteinase K, as well as blocking of endogenous peroxidase by 3% H_2_O_2_. Biotin-labeling of exposed 3′-OH ends of DNA of apoptotic cell nuclei was then performed by adding TdT enzyme and TdT labeling reaction mix for 90 minutes at room temperature, after which the reaction was stopped using the stop buffer. Biotinylated DNA was detected by incubation with a streptavidin-HRP conjugate for 30 minutes at room temperature, followed by a diaminobenzidine (DAB) substrate solution for 15 minutes at room temperature. The samples were counterstained with methyl green, dehydrated and mounted as above. *Ki-67 staining*: Samples were deparaffinized and subjected to a pretreatment with 10 mM citrate buffer, pH 6.0 at 98°C for 10 minutes, as well as blocking of endogenous peroxidase by 1% H_2_O_2_, before incubation with primary detection antibody (rabbit-anti-Ki67 mAb, #ab16667, Abcam, 1:100) overnight at 4°C. Envision-HRP anti-rabbit (#4003, Dako) was employed as a secondary antibody, and NovaRED HRP substrate (#SK-4800, Vector Burlingame) was added as a color substrate for 9 minutes before rinsing and adding a hematoxylin counterstain (HARRIS HTX solution, #01800, HistoLab). Dehydration and mounting of samples were then performed as described above.

### Western blot of tumor samples for uPARAP expression

Tumor samples were crushed to a fine powder in liquid nitrogen, transferred to Eppendorf tubes, and dissolved in lysis buffer (0.1 M Tris, 50 mM NaCl, 1% CHAPS, pH 7.5, including protease inhibitor cocktail III 1:200 (VWR)), using 800 μL of lysis buffer per 150 mg tissue. The tissue was lysed using a chilled polytron tissue homogenizer for 2×20 seconds, or until homogenous appearance. Samples were centrifuged at 12,000 x G at 4°C, and the supernatant transferred to fresh tubes. The protein content was determined by BCA analysis as above. Tissue lysate samples of 20μg total protein per lane were separated by SDS-PAGE and transferred to a PVDF membrane, as described above. For specific detection of uPARAP, mAb 2h9 was labeled with ^125^I [77]. The membrane was then blocked in 2% BSA, and incubated overnight at 4°C with 20 ng/mL of the radiolabeled detection antibody, before being washed in PBS pH 7.4 and water. Using a Fuji FLA-3000 system, an imaging plate was then exposed to the membrane overnight, and developed the following day.

### Statistics

ELISA data are represented as mean of duplicates. All other data are represented as mean ± standard deviation of triplicates. Statistical analysis was performed as a standard t-test.

## SUPPLEMENTARY MATERIALS FIGURES


