# Supplementary material for: The collagen receptor uPARAP/Endo180 as a novel target for antibody-drug conjugate mediated treatment of mesenchymal and leukemic cancers
Source: Oncotarget. 2017 May 16;8(27):44605–24. doi: 10.18632/oncotarget.17883 (PMC5546505; doi:10.18632/oncotarget.17883)
Supplement: Supplementary file 1 [file oncotarget-08-44605-s001.pdf]

# The collagen receptor uPARAP/Endo180 as a novel target for antibody-drug conjugate mediated treatment of mesenchymal and leukemic cancers

## SUPPLEMENTARY MATERIALS

## SUPPLEMENTARY FIGURES

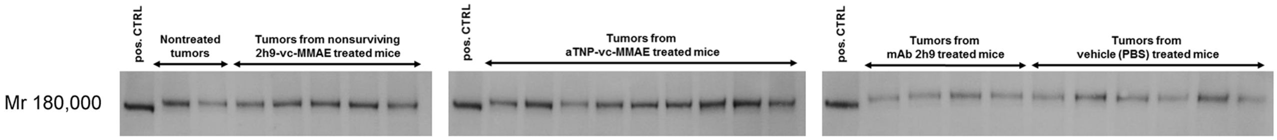


**Supplementary Figure 1: Western blot of total protein harvested from tumor tissue following subcutaneous treatment with 2h9-vc-MMAE (nonsurviving mice), aTNP-vc-MMAE, mAb 2h9 or PBS, using mAb 2h9 for detection of uPARAP.** uPARAP expression was demonstrated in all tissue samples.

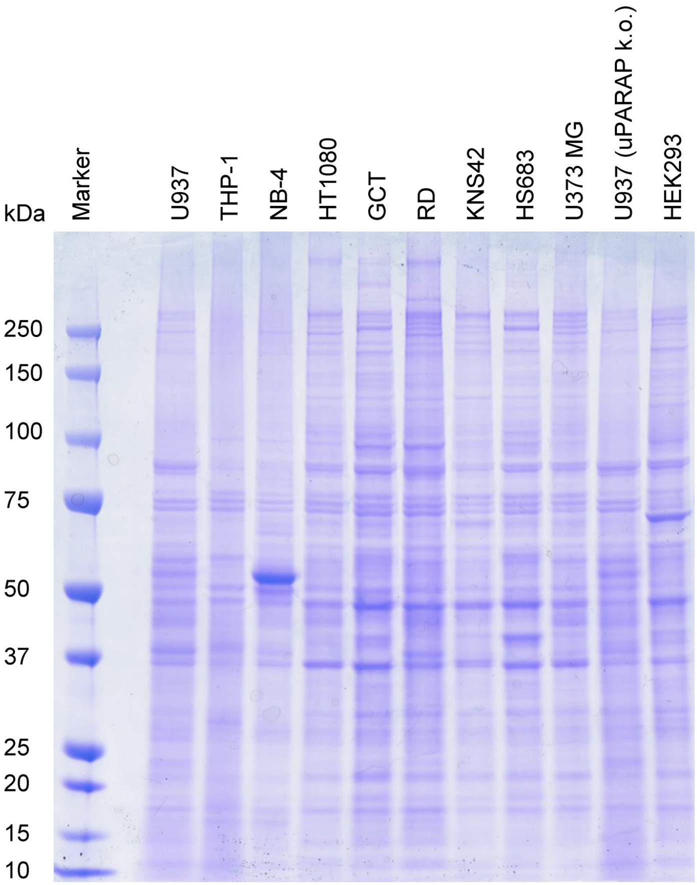


**Supplementary Figure 2: Cell lysates depicted in Figure 1 were further analyzed by SDS-PAGE and Coomassie staining, documenting an over-all uniform protein loading in all lanes.**
